# Supplementary material for: Genomic epidemiology of early SARS-CoV-2 transmission dynamics in Bangladesh
Source: Virol J. 2024 Nov 13;21:291. doi: 10.1186/s12985-024-02560-2 (PMC11562509; doi:10.1186/s12985-024-02560-2)
Supplement: Supplementary file 1 — Supplementary Material 1 [file 12985_2024_2560_MOESM1_ESM.docx]

**Supplementary Appendix**

**
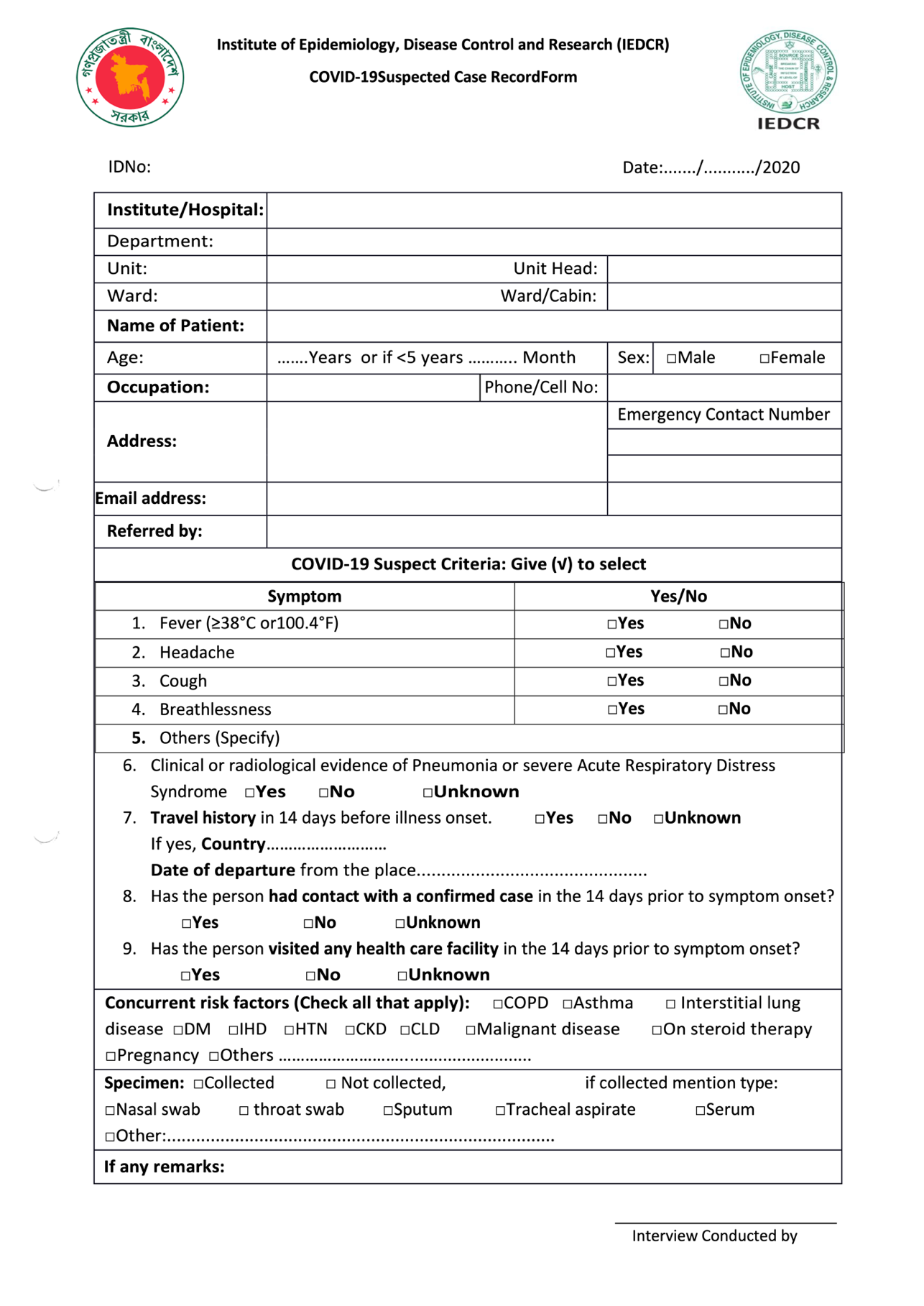
**

***Figure S1.*** *Questionnaire used to obtain epidemiological information from patients alongside swab samples, that were then received by the National Reference Laboratory for Avian Influenza, Bangladesh Livestock Research Institute (BLRI).*


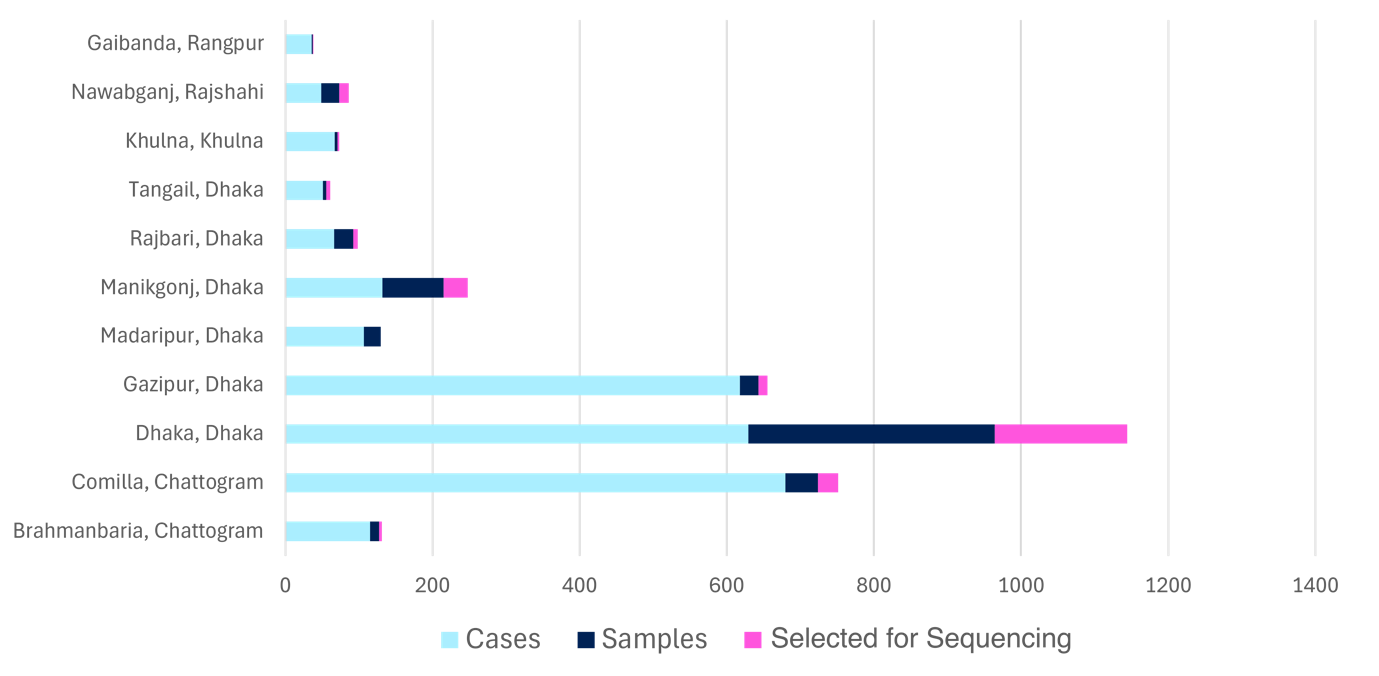


***Figure S2.*** *Selection of samples that were received by the National Reference Laboratory for Avian Influenza, Bangladesh Livestock Research Institute (BLRI) for sequencing. Locations are listed according by district and division.*


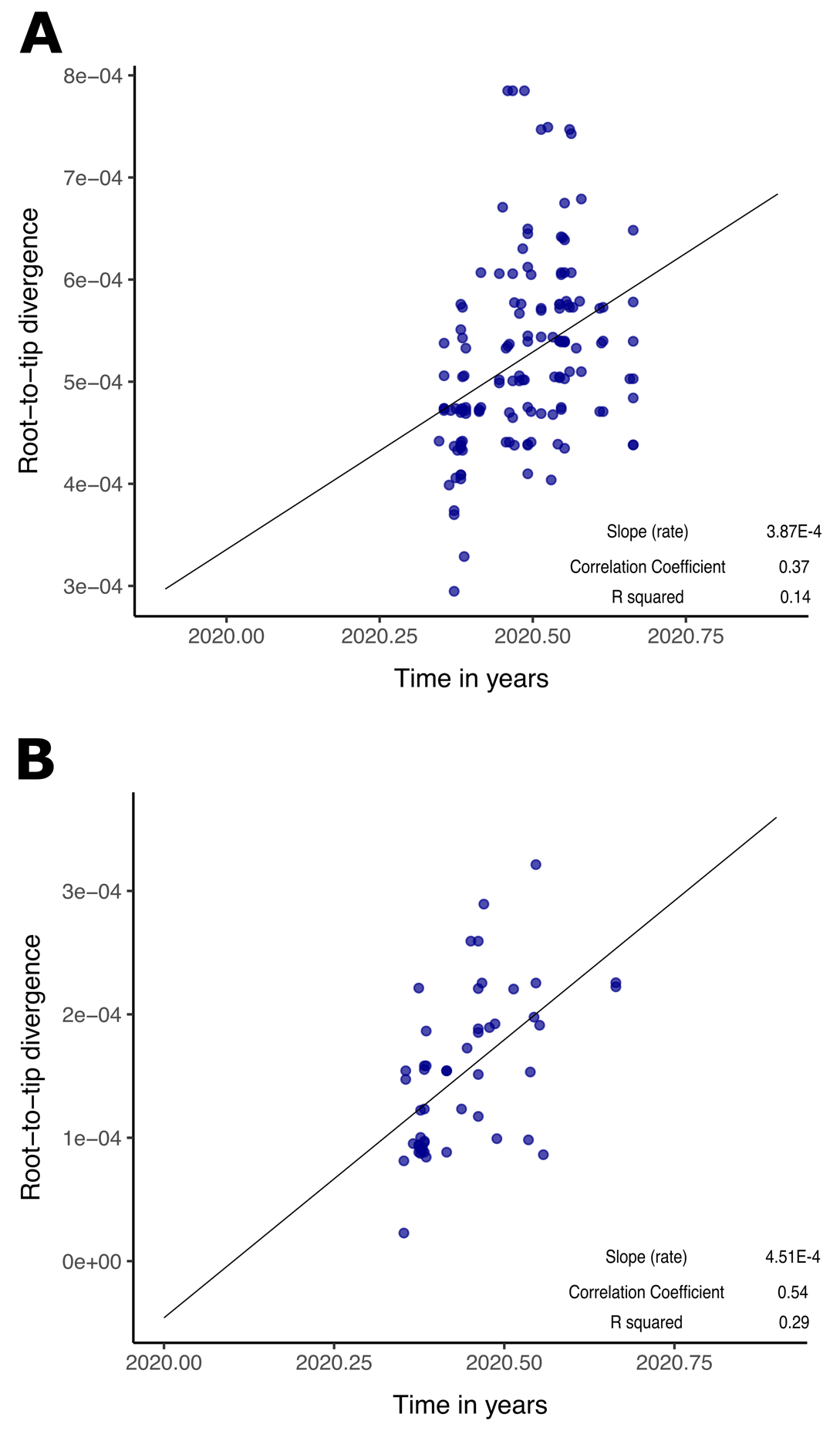


***Figure S3.*** *Root-to-tip divergence plots and the associated statistics for the* ***A****) lineage 2 dataset, and the* ***B****) lineage 8 dataset.*


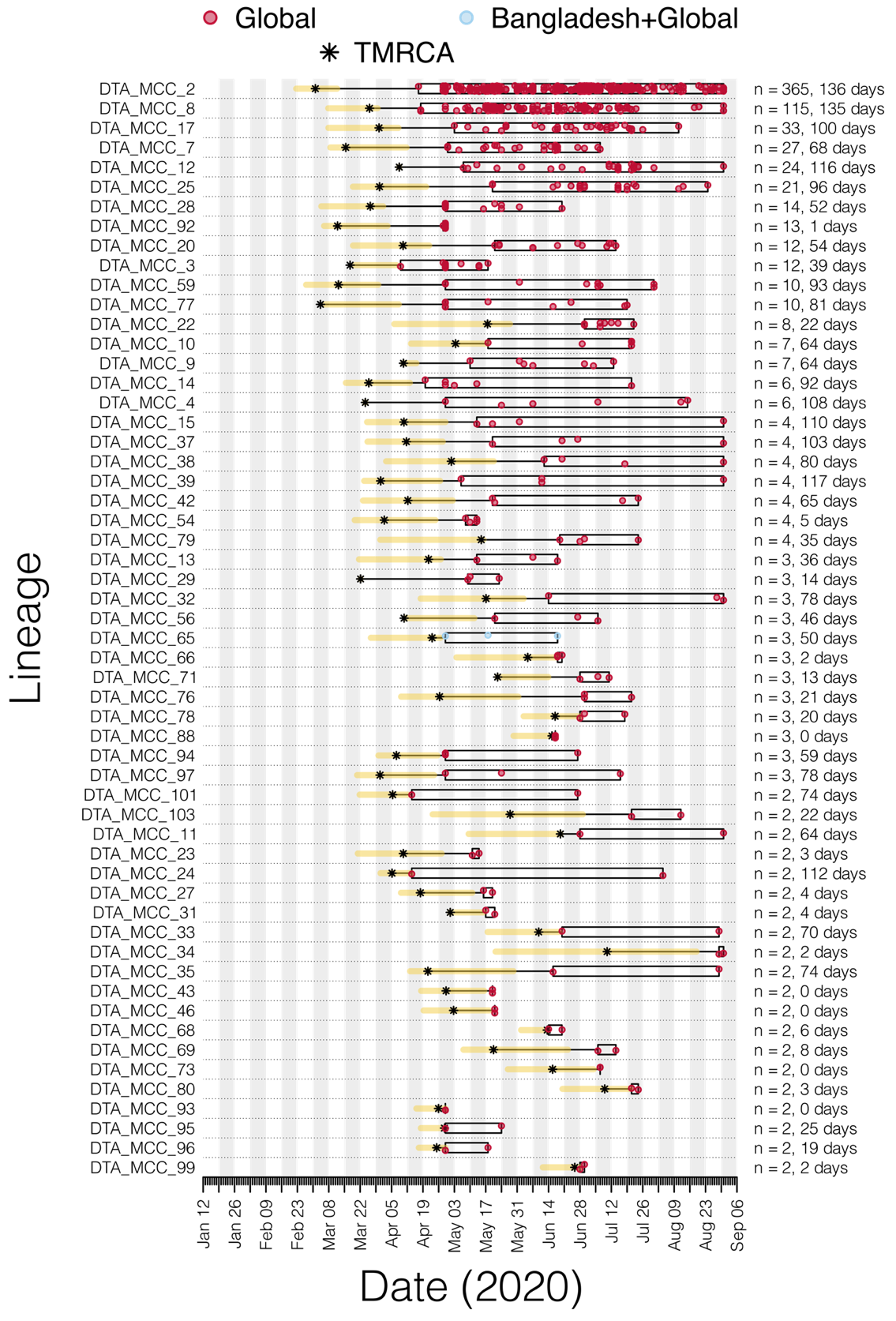


***Figure S4.*** *Duration and timing of Bangladesh transmission lineages. Each row represents a transmission lineage, and red dots indicate genome sampling times. Boxes and labels on the right axis show the sampling duration, and number of sampled genomes (n). Asterisks show the median estimated TMRCA of each lineage, with the 95%* *HPD as a yellow bar.*


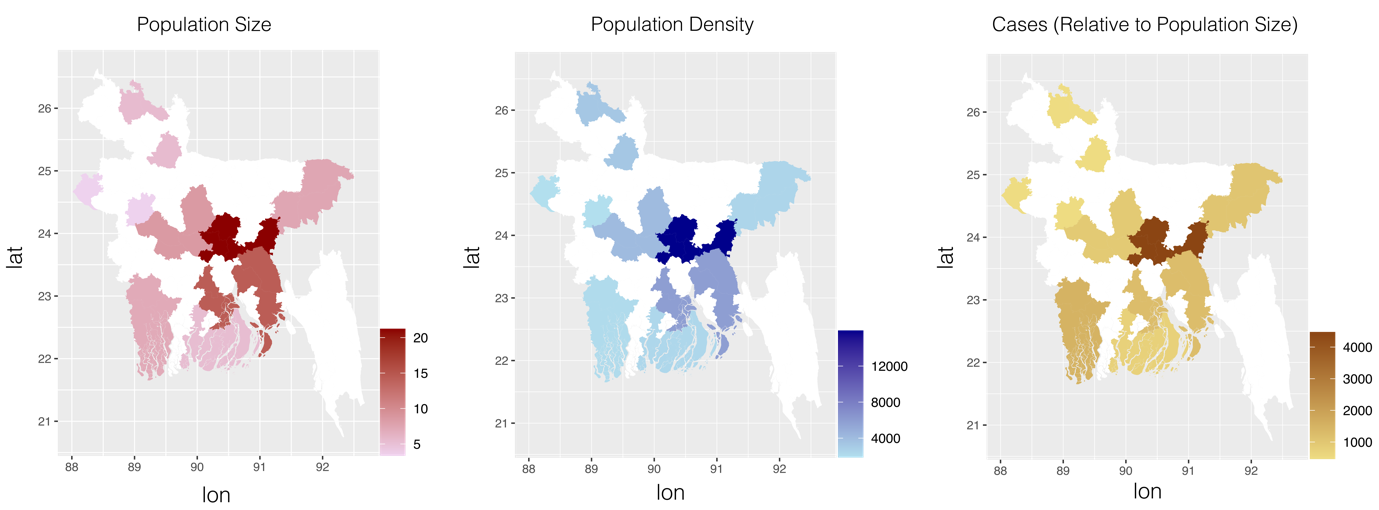


***Figure S5.*** *Choropleth maps for each predictor in the DTA-GLM. Values for each district on the map represent the mean value for the geographic region it belongs to. Regions not represented in this study are in white.*

***Table S1.*** *Total number of sequences per district compared to number of confirmed cases during the sampling timeframe for sequences used in our inter-regional virus spread analyses.*

| **District** | **Number of Sequences** | **Number of Confirmed Cases in Study Period** |
| --- | --- | --- |
| Gaibanda | 3 | 1057 |
| Lalmonirhat | 1 | 734 |
| Nilphamari | 1 | 826 |
| Nawabganj | 1 | 677 |
| Natore | 1 | 893 |
| Manikgonj | 14 | 1354 |
| Pabna | 5 | 1424 |
| Rajbari | 1 | 2791 |
| Tangail | 1 | 2422 |
| Hobiganj | 4 | 1224 |
| Moulvibazar | 9 | 1524 |
| Sylhet | 2 | 5130 |
| Brahmanbaria | 12 | 2315 |
| Dhaka | 48 | 84048 |
| Gazipur | 9 | 3308 |
| Narayangonj | 13 | 5260 |
| Jessore | 6 | 3600 |
| Khulna | 5 | 6147 |
| Shatkhira | 3 | 1024 |
| Comilla | 8 | 6148 |
| Barisal | 3 | 3548 |
| Chandpur | 15 | 2426 |
| Noakhali | 17 | 4406 |
| Madaripur | 3 | 1467 |
| Borgona | 1 | 954 |
| Bhola | 3 | 752 |
| Patuakhali | 3 | 1156 |
| Pirojpur | 2 | 1090 |

***Table S2.*** *List of districts in each geographical grouping.*

| **Geographical Region** | **District** |
| --- | --- |
| Region 1 | Gaibanda |
| Region 1 | Lalmonirhat |
| Region 1 | Nilphamari |
| Region 2 | Nawabganj |
| Region 2 | Natore |
| Region 3 | Manikgonj |
| Region 3 | Pabna |
| Region 3 | Rajbari |
| Region 3 | Tangail |
| Region 4 | Hobiganj |
| Region 4 | Moulvibazar |
| Region 4 | Sylhet |
| Region 5 | Brahmanbaria |
| Region 5 | Dhaka |
| Region 5 | Gazipur |
| Region 5 | Narayangonj |
| Region 6 | Jessore |
| Region 6 | Khulna |
| Region 6 | Shatkhira |
| Region 7 | Comilla |
| Region 7 | Barisal |
| Region 7 | Chandpur |
| Region 7 | Noakhali |
| Region 7 | Madaripur |
| Region 8 | Borgona |
| Region 8 | Bhola |
| Region 8 | Patuakhali |
| Region 8 | Pirojpur |

***Table S3.*** *Number of sequences per geographic grouping (total, and per lineage).*

| **Geographical Region** | **No. Seq.** | **No. Lineage 2 Seq.** | **No. Lineage 8 Seq.** |
| --- | --- | --- | --- |
| Region 1 | 5 | 5 | 0 |
| Region 2 | 2 | 1 | 1 |
| Region 3 | 21 | 14 | 7 |
| Region 4 | 15 | 11 | 4 |
| Region 5 | 82 | 51 | 31 |
| Region 6 | 14 | 13 | 1 |
| Region 7 | 46 | 41 | 5 |
| Region 8 | 9 | 6 | 3 |

***Table S4.*** *GLM Covariate details and sources*

| **Covariate** | **Data Source** |
| --- | --- |
| Population Size (Millions) | Bangladesh Population and Housing Census 2011: <http://www.bbs.gov.bd/site/page/47856ad0-7e1c-4aab-bd78-892733bc06eb/Population-and-Housing-Census> |
| Population Density (100m^2^) | We determined the total area of each region in km^2^ using the area function of raster package. We then calculated the population density of each region by dividing the total area by the total population size (Bangladesh Population and Housing Census 2011: [http://www.bbs.gov.bd/site/page/47856ad0-7e1c-4aab-bd78-892733bc06eb/Population-and-Housing-Census /](http://www.bbs.gov.bd/site/page/47856ad0-7e1c-4aab-bd78-892733bc06eb/Population-and-Housing-Census%20/)). We finally multiplied by 10000 to give values greater than 1, giving a value for population density on a 100m^2^ scale. |
| Mean Daily Confirmed Cases (per Million People) | Institute of Epidemiology Disease Control and Research (IEDCR) “COVID-19 Dynamic Dashboard for Bangladesh, hosted at [http://103.247.238.92/webportal/pages/covid19.php#](http://103.247.238.92/webportal/pages/covid19.php)  We eliminated days where data was missing from any region, and then calculated the mean daily case count for whole study period.  We then divided this mean value by the total population size of the region (Bangladesh Population and Housing Census 2011: [http://www.bbs.gov.bd/site/page/47856ad0-7e1c-4aab-bd78-892733bc06eb/Population-and-Housing-Census /](http://www.bbs.gov.bd/site/page/47856ad0-7e1c-4aab-bd78-892733bc06eb/Population-and-Housing-Census%20/)). |

***Table S5.*** *GLM predictor values for each geographical region (group of divisions).*

| **Geographical Region** | **Population Size (Millions)** | **Population Density (100m^2^)** | **Mean Daily Confirmed Cases (Per Million People)** |
| --- | --- | --- | --- |
| Region 1 | 5.47 | 10.80 | 3.77 |
| Region 2 | 3.36 | 9.22 | 4.77 |
| Region 3 | 8.57 | 10.25 | 7.34 |
| Region 4 | 7.44 | 8.53 | 7.79 |
| Region 5 | 21.23 | 36.01 | 32.64 |
| Region 6 | 7.07 | 7.51 | 12.09 |
| Region 7 | 14.41 | 13.40 | 9.12 |
| Region 8 | 5.32 | 7.75 | 5.71 |

***Table S6****. GISAID EPI SET acknowledgements table for 175 newly published sequences* **(see separate file).**

***Table S7****. GISAID EPI SET acknowledgements table for 103 sequences from lineage 2 accessed from* GISAID [34] epiCOV database ([www.gisaid.org](http://www.gisaid.org)) **(see separate file).**

***Table S8****. GISAID EPI SET acknowledgements table for 37 sequences from lineage 8 accessed from* GISAID [34] epiCOV database ([www.gisaid.org](http://www.gisaid.org)) **(see separate file).**
